# Brain Death and Organ Transplantation in Nepal: Navigating Cultural, Legal, and Ethical Landscapes

**DOI:** 10.3389/ti.2023.11882

**Published:** 2023-11-28

**Authors:** Alok Atreya, Priska Bastola, Swasti Bhandari, Samata Nepal, Prawesh Singh Bhandari

**Affiliations:** ^1^ Department of Forensic Medicine, Lumbini Medical College, Palpa, Nepal; ^2^ Department of Cardiothoracic and Vascular Anaesthesiology, Maharajgunj Medical Campus, Kathmandu, Nepal; ^3^ Lumbini Medical College, Palpa, Nepal; ^4^ Department of Community Medicine, Lumbini Medical College, Palpa, Nepal; ^5^ Department of Orthopedics, Maharajgunj Medical Campus, Kathmandu, Nepal

**Keywords:** organ transplantation, brain death, Nepal, cultural barriers, ethical considerations

## Abstract

Organ transplantation after brain death is challenging in Nepal due to cultural beliefs, legal frameworks, and ethical considerations. The Human Body Organ Transplantation (Regulation and Prohibition) Act (HBOTA) has not met with substantial success after its amendment. This review critically appraises the current state of brain death and organ transplantation in Nepal. It explores challenges, evaluates progress, and provides recommendations. Literature review of databases was conducted to find articles on brain death, organ donation, and transplantation in Nepal. Analysis of cultural, legal, ethical, and practical factors influencing implementation. Key challenges include limited awareness, religious beliefs, infrastructure gaps, and family consent barriers. HBOTA amendments in 2016 enabled brain death donations, however, donation rates remain low. Strategies are needed to improve public education, resources, personnel training, and collaboration. Cultural sensitivity and stakeholder engagement are crucial. A multifaceted approach addressing cultural, legal, ethical and practical dimensions is essential to improve organ donation rates in Nepal. Despite progress, substantial challenges persist requiring evidence-based strategies focused on awareness, capacity building, policy improvements, and culturally appropriate community engagement.

## Introduction

Organ transplantation after brain death diagnosis remains challenging in Nepal due to cultural beliefs, legal frameworks, and ethical considerations. The Human Body Organ Transplantation (Regulation and Prohibition) Act (HBOTA), enacted in 1998 and amended in 2016, aimed to facilitate organ donation and transplantation, but has not achieved expected success [1].

This review focuses specifically on the current landscape of brain death determination and organ transplantation in Nepal. It assesses the multifaceted cultural, legal, infrastructural, and ethical factors that influence the implementation of deceased organ donation programs. The review critically evaluates HBOTA legislation and amendments in terms of their impact on enabling or hindering organ donation rates. It highlights persistent barriers to transplantation in Nepal, including limited public awareness, inadequate health infrastructure, lack of trained personnel, and religious/cultural opposition.

By synthesizing evidence on these challenges, the review offers targeted strategies and recommendations to improve donation rates within Nepal’s sociocultural context. It emphasizes the need for collaborative efforts engaging key stakeholders—government, legal, healthcare, religious, public. The review intends to inform healthcare policies and practices to establish an ethical, effective, and context-appropriate organ transplantation system in Nepal.

This review provides evidence-based recommendations centered on addressing the specific cultural, legal, and ethical barriers to organ donation that are unique to the Nepali context. By providing a comprehensive analysis focused on Nepal’s complex transplant landscape, this review seeks to catalyze improvements that will save more Nepali lives through organ donation.

## Declaration of Brain Death in Nepal

In Nepal, the first organ transplant from a brain dead donor occurred on 8 May 2018. Over the course of approximately 6 years, only 13 people have managed to receive organs from brain dead donors. Despite the introduction of a strong act on brain death in Nepal, expected success has not been achieved due to inadequate implementation measures [1].

According to the Human Body Organ Transplantation (Regulation and Prohibition) Act (HBOTA), the physician shall declare brain death according to Section 12(b) if there is a certainty of brain death in a patient during a health examination, and then the coordination unit should be informed immediately after declaring brain death. After the family approval, a postmortem examination should be performed, and a report should be prepared before proceeding to harvest organs [2]. According to HBOTA Section 12(f), the physician who examines the patient should be a) an MBBS doctor with a minimum of 5 years of experience in the field, b) registered with the Nepal Medical Council as a specialist physician, and c) a citizen of Nepal. According to Section 12(i) of the HBOTA, the doctor-declaring a person brain dead must be someone other than the surgeon performing organ transplantation [3].

In the health institution where the declaration of brain death is made, the following prerequisites must be fulfilled [2]:

a. At least one anesthesiologist or intensivist and a consultant specialist physician.
b. At least two ventilators.
c. An intensive care unit (ICU) with at least two monitors.
d. An operation theater with an anesthesia machine, monitor, ventilator, and electro-surgical unit (diathermy).
e. Facilities for arterial blood gas and computed tomography (CT) scan in the health institution or another local hospital.

Thousands of people are killed in road accidents in Nepal each year, and the number of fatalities has been increasing over the last decade [4]. Many of these unfortunate victims have the potential to become brain-dead organ donors [5]. In a country of about 30 million people, only about 2,100 have agreed to donate organs after brain death. In terms of the disease, this number is considered negligible [6]. Beating heart organ donors are literally nonexistent in Nepal as the beating heart hinders the acceptance by the general population that the person is clinically dead. Those families who reject the brain death of a person with a beating heart insist on keeping them on a ventilator in the belief that they have extended the life of the person. This situation results in the unfortunate misuse of ventilators for futile treatments, which impedes critically ill patients in desperate need of the necessary care and treatment they require [3].

Overall, the HBOTA guidelines need more detailed and flexible clinical, ancillary testing and implementation criteria for successful uptake of brain death diagnosis in Nepal. Based on a comparison of Nepali HBOTA guidelines with international practices on brain death diagnosis [7, 8], here are some key limitations and recommendations:

### Limitations of Nepali HBOTA Guidelines for Brain Death Diagnosis

a. Strict prerequisite of having an intensivist and multiple specialist may not be feasible in many centers, limiting brain death diagnosis.
b. Mandating Nepali citizenship for diagnosing physicians can restrict expertise.
c. No clear minimum observation period stated before testing brain death.
d. No specific guidance on the clinical examination, apnea testing protocols or ancillary tests for brain death diagnosis.
e. No provisions for diagnosis in children or specific circumstances such as trauma, cardiac arrest, etc.
f. Only CT scans are needed for imaging, whereas magnetic resonance imaging (MRI), angiography, and nuclear scans may be needed.
g. Does not address documentation, qualifications, education or legal aspects in detail.

### Recommendations for Improvement

a. Consider minimum observation periods before testing according to international guidelines.
b. Provide detailed guidance on the stepwise clinical examination, apnea testing protocols and ancillary testing criteria.
c. Include special considerations for diagnosis in children and specific clinical circumstances.
d. Allow for wider neuroimaging modalities like angiography, MRI, nuclear scans if needed.
e. Standardize the documentation and qualification requirements for diagnosing physicians.
f. Develop education programs and simulation training in brain death diagnosis.
g. Address legal provisions on time of death, dispute resolution, continued organ support, etc.
h. Relax strict specialty and citizenship requirements to increase the availability of qualified physicians, especially from foreign countries willing to work or volunteer in Nepal.

## Challenges and Barriers for Organ Donation

Multiple obstacles impede the donation of deceased organs in Nepal despite the legal recognition of brain death.

Although major religions endorse organ donation, deeply rooted cultural beliefs in Nepal pose barriers to the acceptance of brain death and deceased donation. For example, in many Nepali communities, bodies are traditionally cremated rapidly after death, reflecting the belief that the soul transitions quickly upon death [3]. Organ donation conflicts with this death ritual. There is also a reluctance to remove organs after brain death since the heart is still beating. Families insist on mechanical life support believing that it extends life, not accepting brain death [9]. Limited public awareness and established cultural beliefs hinder acceptance of brain death. Many Nepalis believe death only occurs after the heart stops beating, resisting organ donation if the heart is still functioning after brain death [3]. Misconceptions that donated organs may be improperly used or family care will be withdrawn also breed distrust. Targeted awareness campaigns are needed, especially in rural communities.

The issue of acquiring organs from deceased individuals becomes a challenge first, in cases involving deceased patients and their families. Second, the family’s consent is crucial for organ donation to proceed. Without explicit consent from the deceased’s family, it would be impossible to proceed with the removal of organs, even if the deceased had previously expressed their consent to be an organ donor. Respecting the wishes and emotions of the family is paramount in such circumstances [10].

A huge gap exists between the demand and supply of organs due to a lack of awareness and a shortage of potential donors. Even when an individual is pronounced brain dead, various obstacles hinder organ donation, including limited awareness, lack of trust and acceptance in the healthcare system, inadequate training of healthcare professionals [5], and incorrect perceptions of brain death.

Inadequate healthcare infrastructure poses barriers to thoroughly assessing and declaring brain death. Diagnostic facilities to conclusively determine brain death are concentrated in major cities [3]. Smaller centers lack ventilators, imaging technology, and specialized medical personnel required by HBOTA to declare brain death. Expanding the capacity of provincial hospitals is essential.

The shortage of trained medical staff also limits the donation processes of deceased. Doctors must receive specialized training to coordinate organ procurement and transplantation according to Nepal’s legal requirements [2], but such training opportunities are limited. More programs are needed to develop this specialized expertise across the country.

## Disparities in Access to Transplantation in Nepal

There are several disparities in access to transplantation in Nepal, including gender, socioeconomic, and geographic disparities. Here are some examples:

### Gender Disparities

Nepal exhibits one of the most extreme gender biases in organ transplantation globally. Among 178 kidney transplants performed at Nepal’s two main transplant centers from 2008 to 2015, 84% of recipients were male while 75% of living donors were female [11]. The majority of organs (65%) were transferred from women to men, while only 6% were transferred from men to women. Mother-to-son donation was the most common (30%), followed by wife-to-husband donation (27%) [11].

This stark disparity stems from deeply gendered social and economic roles in Nepal. Women often feel compelled to donate kidneys to their husbands or sons due to fears of becoming destitute widows or failing their domestic duties [11, 12]. As wives are expected to manage household affairs, women also wish to avoid burdening extended families [11]. Sons represent critical breadwinners and mothers’ parental obligations persist into their children’s adulthood. In contrast, men rarely donate kidneys to female relatives as their livelihoods are not dependent on their wives’ survival [11].

While women assert some agency in choosing to donate, their decisions occur within a patriarchal context that limits autonomy. Legal restrictions on living donation to unrelated individuals exacerbates gender bias by severely limiting women’s donor options [11, 12]. Caste and socioeconomic factors further intersect to shape gendered motivations and perceptions around organ transplantation [11].

Targeted efforts to promote gender equity and men’s donation to women are needed to address this imbalance. Increased public outreach and financial subsidies have been instituted but restrictive legislation continues to constrain women’s access to transplantation in Nepal [11, 12]. Systemic changes transforming women’s societal status and independence are critical to creating an ethical and equitable organ transplantation system.

### Socio-Economic Disparities

Nepal exhibits extreme socio-economic disparities in access to organ transplantation. Due to high costs, transplantation is disproportionately accessed by wealthy socio-economic groups.

A study on 161 kidney transplant patients observed that higher socioeconomic status was associated with better quality of life for transplant recipients [13]. It provides some indication of socioeconomic disparities in access to kidney transplantation in Nepal [13]. Given the high costs and limited availability of transplantation services in Nepal, it is likely more affluent patients are better able to access these treatments [13]. The positive association between socioeconomic status and quality of life outcomes suggests wealthier patients may experience improved wellbeing and recovery after receiving scarce transplantation resources [13]. Although further research is needed, these results imply there may be significant socioeconomic barriers limiting access to organ transplantation for lower income Nepalese patients with end-stage renal disease. Tackling such disparities will be key to ensuring more equitable provision of transplantation services and improving outcomes for economically disadvantaged patients in need of vital organ transplants.

### Caste and Education Based Disparities

Caste also impacts access to transplantation in Nepal. Recipients are disproportionately upper-caste Brahmin and Chhetri groups, likely reflecting greater household incomes. Costs pose major barriers to lower-caste and marginalized indigenous groups accessing transplantation through legitimate channels.

Educational status similarly impacts access to transplantation. Illiteracy rates are higher among lower castes and classes in Nepal. Lack of transplant awareness and inability to navigate complex medical systems impedes illiterate and uneducated patients from obtaining transplants.

### Geographic Disparities

Access to transplantation services is limited in rural areas of Nepal [3]. This is due to the lack of infrastructure and trained human resources in these areas, which makes it difficult to provide transplantation services. The lack of awareness of organ donation in rural areas is another factor that contributes to geographic disparities in access to transplantation [3]. Geographic barriers significantly limit access outside Nepal’s major cities. Few facilities offer organ transplant services in rural areas, and transportation of organs across long distances is logistically difficult [3]. Geographic region further determines access. More than 80% of Nepal’s kidney transplants take place in the capital city of Kathmandu. Fewer centers and nephrologists in rural areas constrain access to transplantation and workup for rural patients. The cost of travel and accommodation to reach the city’s transplant center is prohibitive for poor Nepalis.

## Public Awareness and Education

The prevalence of chronic diseases and end-stage organ damage has been rising. Advancements in medical technologies continue to enhance our ability to diagnose these conditions, intensifying the demand for organ transplantation. Organ transplantation provides the most effective treatment for end-stage organ failure, offering patients an opportunity for healthier living [3]. Comprehensive public awareness campaigns are pivotal to increasing organ donation in society. No major religion explicitly prohibits organ donation, so religious leaders have an important role in advocating its merits among their communities [5]. Educating and informing families about brain death is crucial to overcoming reluctance towards donating organs [14].

In Nepal, successful promotion of organ transplantation requires multifaceted awareness strategies to dispel myths, correct misconceptions, and challenge traditional beliefs impeding organ donation. These include targeted campaigns [3], school education programs [15], community engagement initiatives [15], public-private sector collaboration [16], and culturally appropriate education [17]. Collectively, these efforts boost public awareness, address misconceptions, and promote a supportive environment for organ transplantation in Nepal. This comprehensive approach can help narrow the gap between organ supply and demand, saving more lives.

## Legal and Ethical Considerations

**FIGURE 1:**
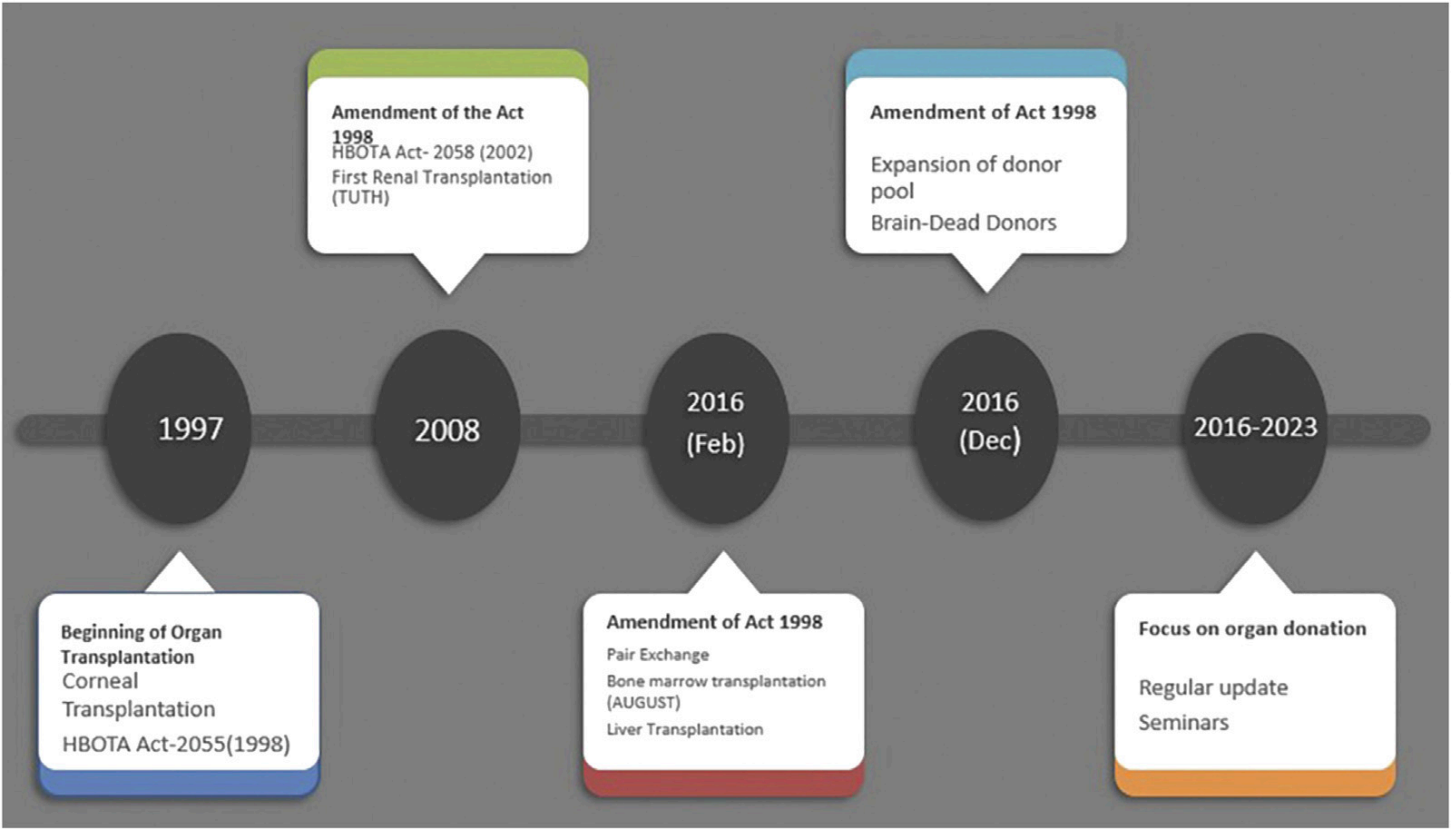
Legislature related to human organ transplantation in Nepal and its amendments.

The 1998 HBOTA law severely restricted organ sales for transplantation, curbing unethical practices. However, it also unintentionally limited organ donations preventing patients with organ failure from receiving transplants even when willing donors were existed. To address this, the HBOTA was amended in 2016, expanding possibilities for organ donation among close relatives [2]. This amendment introduced pair-exchange programs, enabling transplants from clinically deceased donors to recipients in need. The 2016 HBOTA amendment significantly expanded the scope of organ donation and enabled life-saving transplantations (Figure 1) [2].

The revised HBOTA (Section 1a) provides a clear definition of brain death as irreversible loss of brain stem functions. It stipulates that brain death can be confirmed [Section 12(b)1] if a doctor performs two separate examinations 6 hours apart and finds:

a) Irreversible brain damage
b) Absence of brainstem reflexes
c) Absence of spontaneous respiration

Written consent must be obtained from patient’s family before examination [Section 12(b)2]. If unavailable, examination can occur with district administration office oversight [Section 12(b)3].

In situations where the examination is performed by the concerned head of the office, Section 12(b)4 stipulates that other senior doctors from the same institution must also be present. This provision ensures that the examination process is conducted with appropriate oversight and expertise.

According to Section 12(e) of HBOTA, doctors must inform families of examination details and diagnosis. In cases where the family members are unavailable or if the patient is unclaimed, the doctor is required to submit information regarding the diagnosis of brain death to both the concerned district administration office and the Ministry of Health. According to Section 12(i) of HBOTA, the doctor determining brain death cannot be the transplant surgeon, ensuring accountability.

As per Section 17 of HBOTA, it is explicitly prohibited to extract organs from the body of a deceased person and transplant them into another body in a way that interferes with the postmortem findings. This restriction applies specifically to cases where the person’s death is a result of murder, suicide, or occurs under doubtful circumstances.

## Progress After Approval for Brain Death Donation in Nepal

The amendment of the HBOTA act in December 2016 approved organ donation from brain dead donors, marking a significant step in Nepal’s history of organ donation and transplantation [2]. Following this, a single organ transplant center situated in the country’s capital successfully initiated procurement of kidneys from brain dead donors and transplanted them in two patients on 11 May 2017 [15]. Moreover, the same team achieved successful kidney transplants from two additional brain-dead donors, benefiting four recipients. These noteworthy accomplishments contributed to the growth of brain-dead donation and transplantation. However, hospitals equipped with state-of-the-art technology have not been able to perform single brain-dead donation and organ transplantation. The infrastructure and human resources at the human organ transplant hospital provided a strong foundation for the execution of its first kidney transplant from a brain-dead donor, with the availability of skilled doctors and staff performing kidney transplants in a large number since 2012. Nevertheless, other centers regularly performing kidney transplantation with adequate resources for the same have not gained a considerable pace in initiating kidney transplantation from brain dead individuals. The absence of such initiation lies in multiple barriers and challenges faced by the institutions primarily in training, as the HBOTA act emphasizes the need for skilled human resources with a minimum of 6 months of training in the field of organ procurement and transplantation center [2]. One of the biggest hurdles lies in generating funding to train a good number of human resources and other skilled personnel.

It has been repeatedly emphasized the growing need for organs for the ever-rising number of organ failures (kidney, liver, heart, lungs), who are denied the potential resource of organs from brain dead donors available in our country are being overlooked.

## Improving Infrastructure and Resources

Organ transplantation is based on regional or national allocation programs for the efficient coordination, fair distribution of organs, logistical, and laboratory support. The immediate allocation of organs to donor identification is essential due to limited storage time. Proper transport and storage facilities are vital to maintain organ viability and ensure optimal conditions for successful transplantation. These facilities are essential for a well-functioning organ transplant system [18]. Hence, it has become essential to improve the infrastructure, allocate resources and facilities for the prompt diagnosis and effective management of organ donation from deceased patients. Currently, only one center has successfully performed kidney transplantation from brain dead donors.

The HBOTA act of Nepal has elaborated on the requirements of any hospital in Nepal that seeks permission for the donation and transplantation of deceased organs [2]. More specifically, the Act ensures the availability of equipment required for transplantation of kidney and liver by the anesthesiologists and surgeons in the operative room and the postoperative room, e.g., facilities for monitoring, dialysis, mechanical ventilator, and color Doppler [2]. The human resources requirements include qualified anesthesiologist, surgeon, nephrologist, gastroenterologist, radiologist, cardiologist, cardiothoracic surgeon, and pathologist. However, the act addresses organ donation, transplantation infrastructure, and human resource requirements for kidney and liver [2].

The current state of tertiary care center hospitals in Nepal, both at the governmental and some private levels, have met the required infrastructure; however, the skilled human resources to perform deceased organ donation and transplantation of organs other than kidneys remain undertrained. Proper planning of infrastructure usage and trained human resources capable of working from the level of notification of declared deceased donors to successful transplantation of organs need rigorous planning and implementation.

Studies have highlighted the unavailability of proper infrastructures as an important barrier to the execution of brain-dead organ donation and transplantation in developing countries [19].

## Collaboration and Networking

It is essential to develop a well-developed networking and collaborative effort at two main levels. First, an efficient system of informing organ procurement organizations (OPO’s), often referred to as the OPO’s, upon arrival of patients in the emergency department, or in the intensive care unit who are potential donors of organs. Second, further communication of the OPO’s to the Organ Procurement Transplantation Network (OPTN) [20].

In the context of Nepal, the establishment of HBOTA Act 2073 has legalized organ procurement and transplantation from brain dead donors, however, the establishment of a well-developed network of organizations working for its implementation is lacking. Hence, the need to develop organizational bodies that can strengthen networking with other organizations working at the state level to smoothen the organ donation and transplantation process has become essential. Functioning of such bodies in our neighboring country India, that collaborates with the national government to develop and implement standardized procedures, oversee transplant operations, ensure the maintenance of a centralized database for organs and transplants, promote organ donation, and educate staff members involved in organ donation, has been able to achieve better results in organ transplantation [21].

One of the recent plans of the Nepali government is to implement organ donation and transplantation in the seven provinces of Nepal through improved collaboration and networking with the National Transplant Center [22]. Working models developed in countries with higher success in organ donation and transplantation, with well-established networking within the hospital Intensive care units regularly audited for reformation and improvisation of the services, can be studied by developing countries like Nepal [20]. However, sociocultural and financial constraints may limit its implementation.

Countries in their early stages of implementation of brain death acts, governments’ support is essential for the achievement of various tasks, such as providing training for transplant coordinators, establishing nationwide networks for organ transplants, implementing fair systems for organ allocation, and promoting the voluntary declaration of intent to donate through organ donation cards, driver’s licenses, and insurance documents [23].

At the level of hospitals, studies have suggested poor coordination among the transplant team within the hospital as one of the barriers to successful implementation of brain death organ donation process [19]. Therefore, it becomes essential to develop trained human resources who can effectively perform organ procurement and transplantation. One of the needs highlighted in the recent international-level meetings carried out in Nepal emphasized the proposal for establishing a reputable institution dedicated to training specialists in the field of transplantation.

## Overcoming Cultural and Religious Barriers

The cultural practices of people in Nepal are heavily influenced by their existing religious beliefs. Respect and the sacred belief of individuals toward the human body, more specifically when in a state of illness, has strongly limited the acceptance of organ donation by the public. It is imperative and holy to accept bodies in an intact state after the death of individuals, which is supported by different religious beliefs [23]. It is important to make the public aware that organs from potential donors are not removed from patients for organ transplantation. The concept of ‘dead donor rule’ plays a vital role in preserving the rights of intended donor patients who are not denied optimal care to save their lives. Therefore, the rule emphasizes that donors are first declared dead before the organ procurement process is initiated [20]. The cultural belief of the public has limited their acceptance of organ donation after the death of their closest ones as factors like essential care being denied, along with other beliefs of organs being abused, misused, and misappropriated [7, 19]. Hence, organ donation from brain dead patients must be made culturally, ethically, and legally acceptable, by maintaining public trust at each step, emphasizing counseling with the patients near ones by the doctors taking care of their near ones in the intensive care unit.

To overcome the cultural and religious barriers in initiating and implementing brain death organ donation, educating and informing the public can be implemented through educational initiatives aimed at improving public awareness and fostering a positive mindset towards organ donation through well-designed public campaigns [23]. The influence of religious leaders, eminent figures, and media influences holds a powerful ability to raise awareness among the public [7, 24]. In Nepal, the approval of organ donation after brain death has been declared by the current Prime Minister of Nepal to inspire acceptance of it by the public [25]. However, the importance of unplanned media coverage of transplant-related stories should not be overlooked. By showcasing patients’ appeals for organs and sharing stories of successful transplants, media coverage can generate public support and enhance trust in the transplantation process [23].

The need to identify the awareness and willingness to donate organs among the public through research at state levels will provide a strong base to plan activities to raise awareness among the public and raise acceptance of the brain death organ donation process [26]. The lack of government funding for such research has slowed down the process of identifying factors hindering the acceptance of public to brain death organ donation.

## Evidence-Based Strategies to Increase Deceased Organ Donation in Nepal

### Public Education and Awareness

• Culturally-targeted education campaigns are needed, especially in rural communities, to correct misconceptions about brain death and promote organ donation acceptance. Formative research identifying knowledge gaps and cultural barriers can inform campaign design.
• Collaborations with religious leaders and strategic media engagement offer opportunities to gain wider public support for organ donation across diverse communities.
• School health programs and community outreach providing brain death and organ donation education represent potential strategies based on success in other countries.
• Controlled studies are warranted to identify optimal public education approaches and quantify impacts on organ donation rates.

### Healthcare Infrastructure and Training

• Expanding diagnostic facilities and building specialized medical expertise in provincial hospitals are essential to increase capacity for brain death determination and organ procurement across Nepal.
• Standardized training programs focused on the complex process of deceased donation are needed to develop skilled coordination teams and transplant personnel aligned with international guidelines.
• Healthcare collaborations can facilitate knowledge transfer and share best practices in deceased donation processes.

### Legal/Policy Reform and Organ Allocation

• Refining Nepal’s brain death legislation to integrate clinical diagnostic criteria from established international guidelines.
• Government-led initiatives to develop organ sharing networks across all provinces to help address geographic disparities.
• Establishing transparent organ allocation policies and oversight mechanisms to countering public distrust and perceptions of organ misuse.
• Further research into gender, socioeconomic, and cultural norms influencing organ access to provide a guide for legislative reforms.

## Conclusions

This article critically reviews various factors that determine brain death and organ transplantation in Nepal. Strategies to account challenges and barriers, such as limited awareness, religious beliefs, and family consent issues are needed. Collaboration, networking, and skilled human resources are crucial to advance organ transplant practices.

Promoting culturally sensitive approaches to guide cultural and ethical consideration, engaging religious leaders and the media are essential for public acceptance of organ donation after brain death. Recommendations for public awareness campaigns, infrastructure developments, and increased collaboration among healthcare centers offer possible strategies to improve transplantation rates in Nepal.

A multifaceted approach that addresses cultural, legal, and ethical dimensions is needed to develop a sustainable and effective organ donation system in the country. Successful implementation of such measures will improve healthcare, and demonstrate Nepal’s commitment to saving lives through organ donation, and support global efforts to reduce the burden of organ failure worldwide.
